# Regional White Matter Damage Predicts Speech Fluency in Chronic Post-Stroke Aphasia

**DOI:** 10.3389/fnhum.2014.00845

**Published:** 2014-10-17

**Authors:** Alexandra Basilakos, Paul T. Fillmore, Chris Rorden, Dazhou Guo, Leonardo Bonilha, Julius Fridriksson

**Affiliations:** ^1^The Aphasia Lab, Department of Communication Sciences and Disorders, University of South Carolina, Columbia, SC, USA; ^2^Department of Psychology, University of South Carolina, Columbia, SC, USA; ^3^Department of Neurosciences, Medical University of South Carolina, Charleston, SC, USA

**Keywords:** aphasia, speech production, non-fluent speech, arcuate fasciculus, uncinate fasciculus, frontal aslant tract, inferior longitudinal fasciculus

## Abstract

Recently, two different white matter regions that support speech fluency have been identified: the aslant tract and the anterior segment of the arcuate fasciculus (ASAF). The role of the ASAF was demonstrated in patients with post-stroke aphasia, while the role of the aslant tract shown in primary progressive aphasia. Regional white matter integrity appears to be crucial for speech production; however, the degree that each region exerts an independent influence on speech fluency is unclear. Furthermore, it is not yet defined if damage to both white matter regions influences speech in the context of the same neural mechanism (stroke-induced aphasia). This study assessed the relationship between speech fluency and quantitative integrity of the aslant region and the ASAF. It also explored the relationship between speech fluency and other white matter regions underlying classic cortical language areas such as the uncinate fasciculus and the inferior longitudinal fasciculus (ILF). Damage to these regions, except the ILF, was associated with speech fluency, suggesting synergistic association of these regions with speech fluency in post-stroke aphasia. These observations support the theory that speech fluency requires the complex, orchestrated activity between a network of pre-motor, secondary, and tertiary associative cortices, supported in turn by regional white matter integrity.

## Introduction

Growing evidence suggests that the integrity of regional white matter constitutes a crucial factor related to preservation of speech fluency in some neurological diseases. Speech fluency, described as either as fluent or non-fluent, is one of the primary behavioral characteristics used to classify aphasia into its subtypes. Non-fluent speech is characterized by short, often effortfully produced and agrammatic utterances, whereas fluent speech (in neurologically intact individuals) is produced with a normal phrase length and prosodic intonation (Albert et al., 1981). Previously, studies localizing speech fluency have focused on the cortical areas involved, attributing speech production to left-hemisphere anterior cortical areas (i.e., frontal operculum: Alexander et al., 1990; Fox et al., 2000; left anterior insula: Dronkers, 1996; and cortical motor areas: Seddoh et al., 1996; Graff-Radford et al., 2014).

Further investigation into connections between these regions has provided information regarding the importance of white matter pathways in clinical ratings of speech fluency, as more contemporary research has focused on the structural connectivity of cortical networks (Naeser et al., 1989; Bates et al., 2003; Catani et al., 2005, 2013; Ogar et al., 2005; Dronkers et al., 2007; Saur et al., 2008; Rolheiser et al., 2011; Fridriksson et al., 2013). The dual-stream model of speech processing (Hickok and Poeppel, 2007) assigns the role of speech production to a dorsal network, and perception process to a ventral network. Although cortical areas pertaining to these processes have been described [see Hickok and Poeppel (2004, 2007)], less is known about specific white matter connections subserving these areas, and whether certain white matter tracts are more crucial predictors of fluency than others. Here, we adjudicate findings from two studies (Catani et al., 2013; Fridriksson et al., 2013) that have implicated different white matter tracts in speech fluency and relate these tracts to current models of speech processing (Hickok and Poeppel, 2007).

Specifically, the relationship between a newly described frontal white matter region, the aslant tract, and decreased speech fluency in patients with primary progressive aphasia (PPA) was reported by Catani et al. (2013). The aslant tract connects the pars opercularis of the inferior frontal gyrus with the pre-supplementary motor area. Given its anatomical location, it has been postulated that this white matter region exerts a role in integrating motor regions within the frontal speech production network (Catani et al., 2012, 2013).

Conversely, a recent study by our group (Fridriksson et al., 2013) investigating the relationship between white matter integrity and fluency demonstrated that damage to the anterior segment of the arcuate fasciculus (ASAF) was the strongest predictor of non-fluent speech. When the uncinate fasciculus was included with the ASAF in this analysis, predictive power significantly increased, suggesting that the uncinate fasciculus may play a complementary role in fluency. When controlling for potentially influential factors such as lexical processing, diadochokinetic rate, and matrix reasoning, the ASAF remained the best predictor of speech fluency.

The studies from Catani et al. (2013) and from our group (Fridriksson et al., 2013) provide converging evidence that white matter damage is a significant determinant of speech impairment. However, the aslant tract has only recently been described (Catani et al., 2012), and the ASAF and aslant tract have not been directly compared in a single study. Therefore, whether one tract is more important for speech fluency than the other remains uncertain, especially in clinical populations. Additionally, Catani and colleagues investigated patients with a unique form of dementia – PPA – with a largely distinct pathophysiology compared with the localized neurological damage after stroke. Hence, it is unclear if the integrity of the aslant tract is also relevant to other forms of aphasia, specifically stroke-induced aphasia, especially since prior research (e.g., Jefferies and Lambon Ralph, 2006; Hodgson and Lambon Ralph, 2008; Jefferies et al., 2008; Tsapkini and Hillis, 2013) has suggested behavioral differences between these two clinical populations. This is an important question since it is largely unknown how plasticity influences restructuring of the language network after regional brain damage. If the aslant tract is a significant predictor of speech fluency, intra-frontal lobe connections may support fluency, and preservation of speech production abilities may not exclusively depend upon inferior frontal to temporal lobe connections (c.f. Guenther, 2006; Hickok and Poeppel, 2007). We hypothesize that damage to the ASAF and damage to the white matter region involving the aslant tract exert an independent, but synergistic, effect on reduced speech fluency in patients with post-stroke aphasia. The current study tested this hypothesis by investigating the relationship between speech fluency (measured through a comprehensive speech evaluation) and the integrity of regional white matter regions (quantified from high-resolution magnetic resonance imaging – MRI) in a large cohort of chronic post-stroke patients with aphasia.

## Materials and Methods

### Participants

This study included 76 patients (32 female, 73 right-handed) with chronic aphasia who have undergone language testing and MRI studies at the University of South Carolina’s Aphasia Lab over a follow-up period of 10 years. Individuals were included if they had a history of one single, left-hemisphere ischemic stroke leading to neurological symptoms. Medical records and acute scans were reviewed to ensure all patients had aphasia secondary to ischemic stroke in order to study a homogenous population. None of the patients had a history of other cerebrovascular diseases or developmental language abnormalities. None of the patients included demonstrated atypical degrees of leukopathy, beyond what is typical for their age range, as ruled by a neurologist who reviewed all scans. Patients involved in this study were no longer participating in active speech therapy due to the chronic nature of their aphasia. Although some patients participated regularly in stroke support groups, speech therapy was not a confounder for our results.

The mean age of the patients was 61.35 years (SD = 12.67, range = 36–83), and the mean time post-stroke was 35.57 months (SD = 47.56, range = 6–276). The University of South Carolina Institutional Review Board approved this study. All subjects agreed to participation and signed an informed consent.

### Language testing

All patients were assessed by one of two speech–language pathologists with at least 15 years of experience with aphasic patients. Behavioral testing for purposes of the current study included the Western Aphasia Battery-Revised (WAB-R) (Kertesz, 2007) and the Pyramids and Palm Trees Test (Howard and Patterson, 1992). To assess speech fluency, as rated on the WAB-R speech fluency subtest, patients completed a verbal picture description task, and the clinician assigned a fluency score that best described each patient’s speech production. WAB-R fluency scores are chosen based on an 11-point scale, where 0 indicates very limited speech production or mutism, and 10 indicates no impairment. Each score corresponds to a description of speech fluency, ranging from short, telegraphic utterances (non-fluent speech), speech output characterized by hesitations, to fluent speech characterized by normal syntactic structure. This scale captures a range of aphasic speech fluency characteristics, while also ensuring reliability and validity in rater assignment. Average WAB-R fluency subtest score was 5.59 (SD = 3.24, range = 0–10). The Pyramids and Palm Trees Test (Howard and Patterson, 1992) served as a non-verbal measure to evaluate semantic processing. Pyramids and Palm Trees Test scores were available for 57 of the 76 individuals; the mean score was 46 of 52 possible (SD = 5.25, range = 26–52). Distribution of aphasia types as classified by the WAB-R was as follows: anomic = 32; Broca’s = 23; conduction = 5; global = 6; transcortical motor = 1; Wernicke’s aphasia = 7. Notably, two patients scored non-aphasic according to the WAB-R aphasia classification cutoff score of 93.8; nevertheless, these patients still demonstrated some persistent deficits.

### MRI data collection

All subjects underwent a structural brain anatomy assessment using a high-resolution T1-MRI performed at least 6 months after the stroke, in close chronological proximity with the language testing session (usually within the same day, but no longer than 1 month apart).

Magnetic resonance imaging data were acquired using a Siemens 3-T Trio System with a 12-channel head-coil located at the University of South Carolina. All patients underwent scanning that included two MRI sequences: (1) T1-weighted imaging sequence using a MP-RAGE (TFE) sequence with a FOV = 256 mm × 256 mm, 160 sagittal slices, 9° flip angle, TR = 2250 ms, TI = 900 ms, and TE = 4.2 ms. (2) A T2-MRI for the purpose of lesion-demarcation (included as a mask for image normalization) used a 3D SPACE (sampling perfection with application optimized contrasts by using different flip angle evolutions) protocol with the following parameters: FOV = 256 mm × 256 mm, 160 sagittal slices, variable flip angle, TR = 3200 ms, TE = 352 ms. The same slice positioning and angulation was used as with the T1 sequence.

#### Preprocessing of structural images

The clinical toolbox (Rorden et al., 2012) for Statistical Parametric Mapping (SPM8; Friston et al., 1995) was used for spatial normalization of images. The spatial normalization process involved a cost-function approach (Brett et al., 2001), which employed binary lesions corresponding to the areas of the necrotic tissue after the ischemic stroke. Lesion masks were demarcated manually on T2 images using MRIcron. T1, T2, and lesion images were co-registered, and T1 images were bias corrected, segmented, and normalized to the 1 mm isotropic ICBM standard brain template using the cost-function approach (Andersen et al., 2010). Modulated adjustment of image intensity was used during spatial normalization. The following parameters were used for preprocessing: (1) [2 2 2 4] Gaussians per class; (2) 60 mm Bias FWHM; (3) very light regularization; (4) 3 mm sampling distance; and (5) trilinear interpolation.

Once T1 images were normalized into standard stereotaxic space, we quantified the voxel-based T1 intensity *Z*-score for each voxel in the left hemisphere, in relationship to the mean and SD of the voxel-based T1-signal intensity from the right hemisphere (restricted by a brain mask including only gray and white matter tissues). T1-signal intensity in white matter regions was used to relate cortical damage (seen as hypointense signal on T1-MRI) to behavior (Tyler et al., 2005). This method may provide better prediction of the relationship between MRI findings and behavioral results when compared to the use of binary lesion classification (Tyler et al., 2005), since it permits the continuous assessment of T1-based integrity of the lesioned hemisphere, i.e., the spatial analysis is not restricted to the lesion areas. This approach employs a continuous quantity of brain damage (image intensity) to predict a behavioral measure (speech fluency). The use of continuous signal change (hypointensities) may more accurately identify lesioned tissue by accepting a range of intensity changes as damaged, based on a referent determined by signal strength in non-damaged tissue (Tyler et al., 2005; Seghier et al., 2008). The rationale behind this approach is that it allows analyses to be based on two continuous variables (hypointensities and speech fluency scale scores), therefore, increasing statistical power and accuracy of variance calculations (Tyler et al., 2005; Royston et al., 2006). We accounted for inter-individual differences in overall signal intensity by calculating *Z*-scores on a voxel-by-voxel basis for each patient’s T1-MRI based on the mean and SD of right hemisphere image intensity of all in-brain voxels using a custom code created in Matlab (The Mathworks, 2012 Natick, MA, USA). All images were normalized to stereotaxic space, enabling a voxel-by-voxel comparison with the contralateral hemisphere. This allowed for standardization based on image intensity of gray and white matter of the intact (right) hemisphere.

#### Region of interest analyses

For each subject, we assessed the mean T1-signal intensity *Z*-score within the areas defined by the following white matter regions selected as region of interests (ROIs): the ASAF, the uncinate fasciculus, and the white matter corresponding to the aslant tract. To ensure that damage to regional white matter ROIs, rather than white matter damage in general, was related to reduced fluency in aphasia, a white matter ROI thought to be unrelated to speech production, the inferior longitudinal fasciculus (ILF) was included as another ROI. The ILF was chosen due to its likely (Duffau et al., 2013) location in the ventral (semantic) stream of language processing (Hickok and Poeppel, 2007; Mandonnet et al., 2007; Duffau et al., 2013). This tract connects occipitotemporal areas to the temporal pole, and is thought to be an ancillary pathway that communicates semantic information through the uncinate fasciculus (Vigneau et al., 2005; Mandonnet et al., 2007). It was therefore predicted that it would not significantly account for fluency scores, but may play a role in semantic processing.

**Figure 1:**
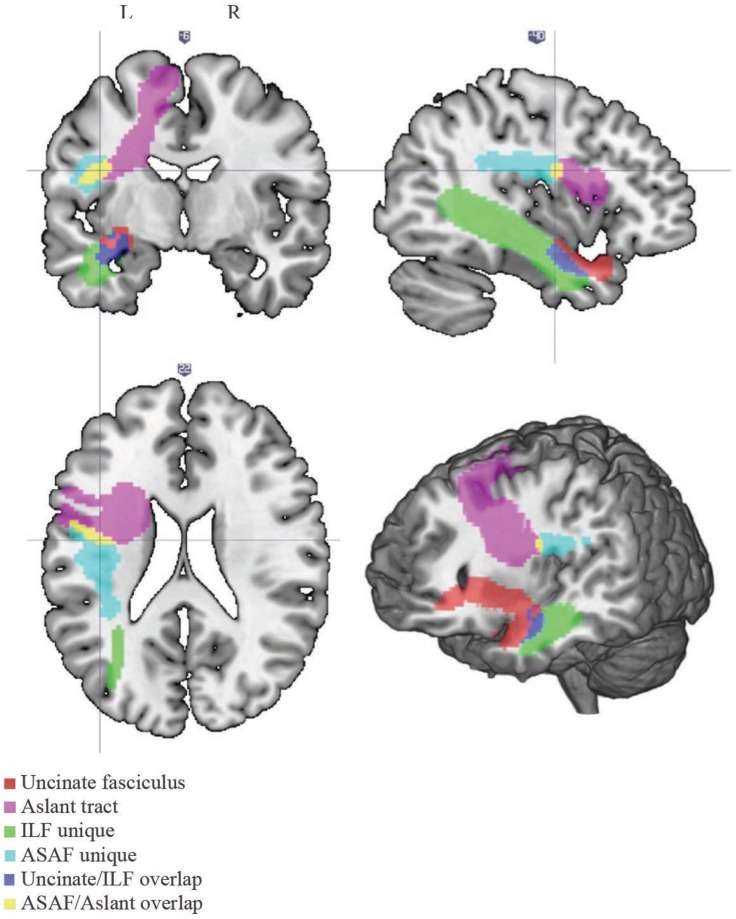
**White matter tracts**. Locations of each of the white matter tracts included in our analyses. Note the following color scheme: magenta – aslant tract; cyan – ASAF; red – uncinate fasciculus; yellow – ASAF/aslant overlap; green – ILF; blue – uncinate/ILF overlap. The ASAF forms a connection between the *pars opercularis* and lateral middle and inferior precentral gyri to the posterior, inferior parietal lobe. The aslant tract connects Broca’s area to pre-supplementary motor areas. The ASAF/aslant overlap underlies BA 6. The uncinate fasciculus connects the anterior and superior temporal lobe to the medial and lateral portions of the inferior frontal cortex. The ILF connects occipitotemporal regions to the temporal pole. The uncinate and ILF overlap in white matter of the inferior temporal lobe (Brodmann area 20) and parahippocampal cortex (Brodmann area 36).

A white matter atlas constructed by Catani et al. (2012) was consulted to define the ASAF, ILF, and the uncinate fasciculus. The ROI used for the aslant region was obtained from Catani and colleagues. Due to notable overlap between the aslant and ASAF and the uncinate and ILF, we created two additional ROIs composed of the areas of overlap between the aslant and ASAF regions (herein referred to as the ASAF/aslant region overlap) and the uncinate fasciculus and ILF (herein referred to as the uncinate fasciculus/ILF overlap). This allowed for analyses based on unique white matter ROIs, as well as ROI overlap. See Figure 1 for locations of each the six ROIs.

Relationships between mean T1-MRI intensity values for each ROI were examined in several ways, both independently, and as part of a unified model. First, we used separate Pearson correlations for each ROI, to predict fluency (WAB-R fluency subtest score) and semantic processing (Pyramids and Palm Trees Test). Next, a Hotelling–Williams *t*-test (Steiger, 1980) was used to compare the strength of each correlation to determine if one ROI was a significantly better predictor of non-fluent speech than the other ROIs. Finally, these intensity values were entered into a stepwise regression analysis including all six ROIs, along with lesion size as a control variable (including both cortical and subcortical damage), to determine which regions remained significant predictors for each behavioral measure, given extent of damage and damage to all other areas as cofactors. Scores on additional WAB-R subtests were not controlled for, as these are highly associated with our dependent variables, fluency, and semantic processing. SPSS (Version 20, IBM Corp.) was used to conduct correlation and regression analyses, and a custom Matlab code (The Mathworks, 2012 Natick, MA, USA) was used to compute Hotelling’s *t*. To control for multiple comparisons in the correlation analyses, the alpha-level was Bonferroni corrected within each behavioral measure by dividing the alpha-level of *p* = 0.05 by the number of regions examined (six). Thus, the threshold for significance was set to *p* = 0.008. To control for multiple comparisons in the regression analyses, the alpha-level was Bonferroni corrected by dividing the alpha-level of *p* = 0.05 by the number of behavioral measures examined (two). Thus, the threshold for significance was set to *p* = 0.025. Because the Hotelling *t* was an explorational analysis, no corrections were made for multiple comparisons.

## Results

The ASAF/aslant ROI overlap (*r* = 0.64, *p* < 0.001), aslant (*r* = 0.634, *p* < 0.001), ASAF (*r* = 0.428, *p* < 0.001), uncinate fasciculus (*r* = 0.574, *p* < 0.001), and the uncinate/ILF overlap (*r* = 0.296, *p* = 0.005) were correlated with speech fluency scores, whereas the ILF was not (*r* = 0.015, *p* = 0.448). The following Hotelling *t* comparisons reveal that the integrity of the ASAF/aslant overlap showed the strongest relationship with speech fluency [ASAF/aslant overlap vs. uncinate fasciculus: *t*(73) = 1.73, *p* < 0.05; ASAF/aslant overlap vs. uncinate fasciculus/ILF overlap: *t*(73) = 3.35, *p* < 0.01; ASAF/aslant overlap vs. ILF: *t*(73) = 3.03, *p* < 0.005; ASAF/aslant overlap vs. ASAF: *t*(73) = 2.51, *p* < 0.01]. The difference between the ASAF/aslant overlap and the unique aslant ROI trended toward statistical significance: *t*(73) = 1.6, *p* = 0.057. Variance inflation factors (VIF) for both models do not suggest issues of collinearity in the data (VIF for ASAF/aslant Overlap model = 1, VIF for ASAF/aslant overlap and uncinate-unique model = 1.28).

In a stepwise regression model, the ASAF/aslant overlap was shown to be the best predictor of speech fluency, *F*(1,74) = 51.34, *p* < 0.0001, *R*^2^ = 0.40. Integrity of the uncinate fasciculus also emerged as a statistically significant predictor of fluency scores, as this ROI significantly increased prediction power of the model [ASAF/aslant overlap and uncinate: *F*(1,73) = 14.28, *p* < 0.0001, *R*^2^ = 0.49]. The relationship between fluency and the remaining tracts investigated was not found to be significant in this model. Collinearity was not significant in this model (VIF = 1).

**Figure 2:**
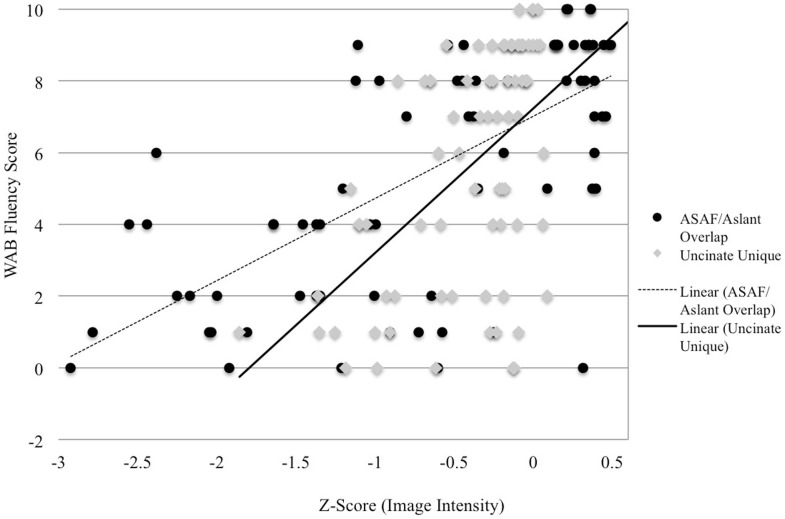
**Image intensity and WAB-R fluency scores**. *Z*-scores for T1-MRI image intensity values for the aslant tract and ASAF (*x*-axis) and WAB-R fluency scores (*y*-axis). Solid and dotted lines depict regression lines of best fit for the uncinate fasciculus and ASAF/aslant overlap, respectively.

**Figure 3:**
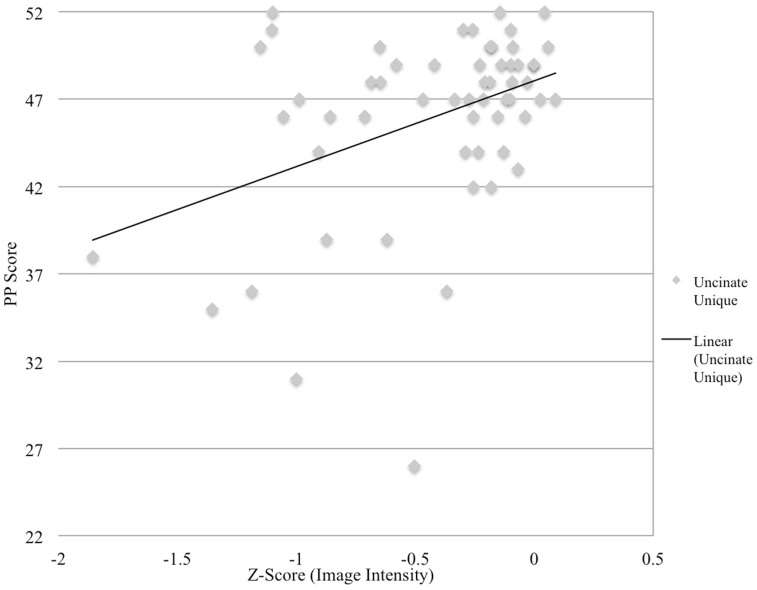
**Image intensity and semantic processing**. *Z*-scores for T1-MRI image intensity values for the uncinate fasciculus (*x*-axis) and pyramids and palms scores (*y*-axis). Solid line depicts regression line of best.

Semantic processing was correlated with the uncinate fasciculus (*r* = 0.41, *p* < 0.001) and the aslant (*r* = 0.37, *p* < 0.008), but none of the remaining ROIs. Correlations for non-significant ROIs are as follows: uncinate fasciculus/ILF overlap: *r* = 0.252, *p* = 0.029, ILF: *r* = 0.22, *p* = 0.051, and ASAF/aslant overlap: *r* = 0.186, *p* = 0.083. There were no significant differences between the strength of each ROI correlations as assessed by the Hotelling–Williams *t*-test (Steiger, 1980). In the stepwise regression model, the uncinate fasciculus was found to the best predictor of semantic processing, *F*(1,55) = 11.3, *p* < 0.001, *R*^2^ = 0.16. None of the remaining ROIs provided additional reduction in variance for predicting semantic processing, suggesting that this tract to be the best predictor of fluency in the model. Results from regression analyses are presented in Table 1, and depicted in Figures 2 and 3.

**Table 1:** **Results from regression analysis**.

Predicted factor	Model	*F*	*p*	*R*^2^	Predictors
Speech fluency	1	51.34	<0.0001	0.40	ASAF/aslant overlap
Speech fluency	2	14.28	<0.0001	0.49	Model 1 and Uncinate-unique
Semantic processing	1	11.3	<0.001	0.16	Uncinate-unique

*Models were selected in a stepwise linear regression analysis. The first column states the predicted factors, the second column states the number of the significant models, the third through fifth columns present model parameters, and the sixth column presents areas that significantly predict behaviors in question*.

Taken together, our findings suggest that overlapping portions of the aslant and the ASAF, along with the uncinate fasciculus, are collectively involved in speech fluency, with the uncinate fasciculus additionally predictive of semantic processing scores.

## Discussion

The current findings further support that regional white matter damage is associated with post-stroke speech fluency. We observed that the integrity of the overlap between two previously reported white matter regions [aslant tract (Catani et al., 2013) and ASAF (Fridriksson et al., 2013)] was a significant predictor of fluency in post-stroke aphasia. We also observed that the uncinate fasciculus is a significant predictor of fluency, which is in accordance with previous observations from our group, suggesting that a supportive role of the uncinate fasciculus in speech fluency as well as semantic processing (Catani et al., 2013; Fridriksson et al., 2013).

Although previous studies have evaluated this topic in the context of other forms of aphasia (Catani et al., 2013), there are relatively few studies comparing the behavioral deficits of PPA and stroke-induced aphasia (Jefferies and Lambon Ralph, 2006; Hodgson and Lambon Ralph, 2008; Jefferies et al., 2008; Faria et al., 2013), with some suggesting qualitative differences in errors between groups (Jefferies and Lambon Ralph, 2006; Hodgson and Lambon Ralph, 2008; Jefferies et al., 2008; Tsapkini and Hillis, 2013). However, when factors such as damage (either frank structural damage or degenerative damage) and time post-onset of stroke (prior to reorganization) were controlled for, Budd et al. (2010) found minimal between groups differences. This suggests that reduced fluency in both disorders may arise from a common neuroanatomical location, but factors such as reorganization (post-stroke) and progressive degeneration (PPA) may modulate language processing differently in both groups over time. Notably, white matter damage in the current study could be attributed to Wallerian degeneration in addition to (or following) cortical gray matter damage (Waller, 1850; Bonilha et al., 2014). However, taken together, the current results and those of the aforementioned studies support that location of damage, rather than etiology, is more important in explaining deficits in these patient groups.

It should be that speech fluency can be measured by different means. For instance, Catani et al. (2012) measured speech fluency as words per minute (WPM), whereas this study employed a standardized clinical measure, the WAB-R fluency subtest. We acknowledge that use of the WAB-R fluency scale is not a perfect measure of speech fluency, as ratings are based on forced-choice assignment on a scale of 0–10 and do not take into consideration how other factors may influence fluent speech production (e.g., speech initiation and maintenance, agrammatism, lexical retrieval). Similarly, WPM does not completely account for other factors that may influence fluent speech production as noted above. Nevertheless, we queried the AphasiaBank database (MacWhinney et al., 2011) to obtain measures of WPM and WAB fluency subtest scores in order to determine the extent that these two fluency measures are related. Scores from 247 individuals were obtained from individuals who had both transcribed speech samples and WAB-R test scores (mean WAB AQ = 70.62, SD = 19.98; mean WAB fluency score = 6.26, SD = 2.482). Indeed, this analysis suggests that the two measures are correlated, *r* = 0.546, *p* < 0.01, with an increase in WPM corresponding to increased fluency scores. This suggests that different measures of fluency are related, but not completely redundant. For purposes of the current study, the relationship between WPM and WAB fluency scores further corroborates the role of the aslant and ASAF in speech fluency, as it has shown to be predictive of fluency in two studies where different (yet related) measures of fluency were utilized.

It should be additionally acknowledged that our study and that of Catani et al. (2013) used different methods for analyzing the white matter regions under investigation. We analyzed image intensity values for each ROI based on neuroanatomical atlases. Analysis of hypointensities allowed us to determine how the degree of structural damage (rather than a binary classification of the presence/absence of damage) influenced performance on a continuous behavioral scale (i.e., performance on the WAB-R fluency scale, the pyramids and palm trees test). This method precludes the need to make forced-choice decisions on lesion boundaries where the difference between necrotic, dysfunctional, and healthy tissue is not always clear. Although T1-signal intensity is an indirect measure of tissue integrity (and is correlated with T2 hyperintensities, also an indirect measure of white matter integrity), T1 images were used as due to better tissue contrast, these scans facilitate more specific regional assessment of damage. The use of T1 images as surrogates for white matter disease has been extensively used in the literature for a variety of neurological disease (either as direct signal measure, or through calculation of voxel-based volume in voxel-based morphometry studies; Paus et al., 2001; Wen et al., 2006; Li et al., 2012a,b; Boddaert et al., 2013).

In contrast, Catani et al. (2013) used whole brain tractography with each tract dissected *in vivo* for each participant, which takes into account individual variability in anatomical structure (Catani et al., 2013). Although clearly different methodologies, both methods allow for the inclusion of patients who may not have damage to specific ROIs, in addition to analyzing the degree of frank tissue damage (our method) or microstructural integrity (Catani’s method) on behavioral performance. We are not able to compare DTI data to those data presented here, nor are we aware of available studies that compare these two approaches. While our results should be interpreted with this caveat in mind, we argue that this approach was adequate in identifying white matter damage of the current patients.

Regardless of these methodological differences, further study of the overlap between the ASAF/aslant and the uncinate fasciculus itself in relation to speech fluency is warranted. The ASAF and aslant overlap in the white matter of Brodmann’s area 6, which is located in the pre-motor cortex and supplementary motor areas. These cortical areas have been suggested to be involved in speech fluency (Fridriksson et al., 2013). Therefore, it is possible that the convergence of white matter regions underlying these cortical regions is crucial for fluent speech production.

Although the mutually exclusive portions of the ASAF and aslant region were significantly correlated with fluency scores, these ROIs were not uniquely predictive of speech fluency. However, all three ROIs represent tracts with connections to and from inferior frontal lobe areas, and the common inferior frontal location of all three tracts argues for the importance of inferior frontal white matter connections in speech fluency. Differences in each tract’s posterior connections indicate that fluent speech production may be served by a widespread cortical network, and the nature of each tract’s locations suggest that connections between inferior frontal and sensorimotor cortical areas serve fluent speech production (Hickok and Poeppel, 2007).

Clearly, each white matter ROI investigated here differs in regard to which cortical areas it connects. The current evidence suggests that speech fluency relies on intact connections between motor and sensory feedback areas, and that the integrity of these tracts may serve these abilities to different degrees. The aslant tract connects inferior frontal areas to anterior and supplementary motor areas, suggesting that its additional role in speech initiation and coordination (Catani et al., 2012). The aslant tract’s front-frontal connections suggest that intra-frontal connections support fluency in addition to frontal–temporal connections traditionally implicated in speech production (e.g., Guenther, 2006; Hickok and Poeppel, 2007). On the other hand, the ASAF serves as a connection between inferior frontal and posterior parietal areas, suggesting that this tract may serve speech fluency by playing a role in auditory feedback loops for integrating sensorimotor information for online monitoring of complex speech production (Hickok and Poeppel, 2007). As evidenced here, damage to the overlapping areas of these tracts may affect the unique processing abilities of each individual tract, negatively influencing each tract’s contribution to speech production. Further investigation regarding how damage to overlapping regions of the ASAF and aslant tract affects possible feedforward or feedback mechanisms of each individual tract for speech production is necessary.

With regard to semantic processing, it has previously been suggested that although the ILF plays a role in semantic processing, damage to this tract may not be necessary or sufficient to lead to a semantic deficit. In a study, where the ILF was transiently disturbed through intraoperative direct electrostimulation or through partial resection, no negative effect was found for performance on a naming task (Mandonnet et al., 2007). It has been suggested that damage to the ILF is readily compensated for, possibly explaining why the ILF was not a significant predictor of semantic processing in the current study. Our results indicate that the uncinate fasciculus was a greater predictor of semantic abilities, which may be explained by this tract’s relationship to semantic hub areas of the anterior temporal lobe (Lambon Ralph et al., 2010).

In conclusion, this work suggests that individual white matter regions may be underappreciated in speech and language, and importantly, that their intersections deserve greater attention in predicting post-stroke speech and language deficits. Moreover, our results add to the debate regarding areas that serve speech fluency, further highlighting the role of white matter connections in speech. Continued investigation of overlap between white matter regions, along with the aslant tract, ASAF, and uncinate fasciculus has the potential to add to our understanding of the symptomology related to stroke-induced and PPA, as well as other neurologic communication disorders. Our current work demonstrates that each of these regions refines our ability to diagnose chronic symptoms. Future studies could explore whether acute scanning to assess the integrity of these connections could be combined with acute behavioral measures to provide an accurate long-term prognosis.

## Conflict of Interest Statement

The authors declare that the research was conducted in the absence of any commercial or financial relationships that could be construed as a potential conflict of interest.
